# Support, networks, and relationships: Findings from a mixed-methods evaluation of a mentorship programme for early career women researchers in sexual and reproductive health and rights

**DOI:** 10.1371/journal.pone.0295577

**Published:** 2023-12-19

**Authors:** Muhammad Asim, Peter Muriuki Gatheru, Joy J. Chebet, Mehr G. Shah, Anna Thorson, Vanessa Brizuela

**Affiliations:** 1 Department of Community Health Sciences, Aga Khan University, Karachi, Pakistan; 2 Population Research Center, University of Texas at Austin, Austin, TX, United States of America; 3 African Population and Health Research Center (APHRC), Nairobi, Kenya; 4 Department of Population, Family and Reproductive Health, University of Ghana, Accra, Ghana; 5 UNDP/UNFPA/UNICEF/WHO/World Bank Special Programme of Research, Development and Research Training in Human Reproduction (HRP), Department of Sexual and Reproductive Health and Research, World Health Organization, Geneva, Switzerland; Nottingham Trent University, UNITED KINGDOM

## Abstract

Low research output among women researchers in health research has been linked to inadequate mentorship opportunities for early career women researchers and particularly in sexual and reproductive health and rights (SRHR) field. Mentorship has been recognized as a contributor to strengthening research capacity and as beneficial for both mentors and mentees. Women researchers oftentimes experience negative impacts of organizational and structural gender inequities related to formal and informal mentoring. In 2020, the UNDP/UNFPA/UNICEF/WHO/World Bank Special Programme of Research, Development and Research Training in Human Reproduction at WHO launched a mentorship programme for early career SRHR women researchers from low- and middle-income countries. The programme sought to provide professional skill-building, promote and share networking opportunities, and offer support in navigating personal and professional life. We conducted a convergent parallel mixed-methods evaluation of the 2020 pilot programme, which included 26 participants, through an online survey and semi-structured in-depth interviews (IDIs). Data collection occurred between March and May 2022. Nineteen responded to the online survey (12 mentees, 7 mentors) and 11 IDIs (7 mentees, 4 mentors) were completed. Based on a preliminary framework, we used deductive and inductive methods to identify six themes: views on mentorship; reasons for applying and expectations of participation in the programme; preferred aspects of programme implementation; challenges with the programme implementation; perceived lasting benefits of the programme; and recommendations for improvement. All participants found the initial training useful, most discussed work-life prioritization throughout the mentorship relationship, and most planned to continue with the relationship. There appear to be ample benefits to mentorship, especially when planned and implemented in a structured manner. These attributes can be particularly beneficial when they are conceived as a two-way relationship of mutual learning and support, and especially for women at the start of their research careers as they navigate structural gender inequities.

## Introduction

Research output from low and middle-income countries (LMICs) particularly from women health researchers continues to lag behind that of high-income countries (HIC). The reasons for this include scarcity of well-trained and skilled researchers resulting in poor supervision of high degree scholars and inadequate career mentorship opportunities for early career researchers [1, 2]. Mentorship opportunities, particularly for women in scientific research in LMICs, are especially scarce [3, 4]. Existing evidence of mentoring programmes for women in science, technology, engineering, and mathematics linked effective mentoring to higher graduation rates compared to women who did not have mentors during their graduate training [5]. While women currently make up 70% of the global health and social workforce, they hold only 25% of senior leadership roles [6]. With the limited number of women in leadership positions or mentor roles, it is challenging for female researchers to access same-gender mentorship [7]. Women researchers have oftentimes expressed experiencing negative impacts of organizational and structural gender inequalities related to formal and informal mentoring [8–10]. Different genders can have varying experiences, in both their personal lives priorities, family commitments and their professional lives as researchers, which may affect the guidance and advice offered by their mentors. Studies highlight that same sex mentor and mentees can mitigate the risk of gender power dynamics influencing the mentor-mentee relationship and structural inequalities [7, 11].

Mentorship has been identified as an important component to strengthen research capacity, and as a tool to empower and promote the professional development of health researchers [12, 13]. While mentoring can be a pathway to increased research output and scientific progress, mentorship programs by women for women researchers are not a common practice in LMICs. Existing mentoring approaches and programs seem to be characteristic of high-income settings that do not take into account the differences in culture, resources and research systems structures in LMICs [4, 14, 15]. Major challenges have been identified related to development of mentorship capacity in LMICs including lack of formal mentorship framework and policies, a well-established mentorship culture, institutional support for mentorship, and scarcity of systematic tools to assess mentorship programs. Other challenges within the health professions include conflict of interest, gender and power imbalance, lack of clarity on concept of mentorship, socio-political contexts and their effects and unrealistic expectations [14–18]. Conversely, benefits of mentoring programs have also been highlighted including providing unique research opportunities for early career researchers, helping the attainment of short-term career goals, development of skills and knowledge, understanding of institutional culture and values critical to the profession [15, 16].

Although many interpretations of mentorship exist in the literature [19–21], there is a general distinction between formal and informal mentorship. Formal mentorship constitutes matching mentees with mentors by a third party as part of an officially sanctioned mentorship program. Conversely, informal mentorship is generally defined as naturally or spontaneously developing relationships that occur without outside interference [22, 23]. Formal mentoring is structured such that mentors and mentees are paired up and assigned to one another with explicit developmental objectives for the mentee [24]. Evidence suggests that formal mentorship programmes can be beneficial for both mentees and mentors, provided the relationship is based on positive values such as a mutual growth and learning [12, 25, 26].

Formal mentorship programmes vary in their design and implementation. Attempts at evaluating these programmes have been challenging due to the inherent *ad hoc* nature of many of them [25, 27]. However, there is sufficient evidence showing that those participating in a mentorship programme require training and skills-building for effective mentoring. Additionally, existing literature indicates that mentorship programmes require clear objectives and operational/logistical set-up to ensure success [27–30]. This includes considerations for institutional support in the form of specific allocated time and resources to provide mentorship as well as an enabling environment for mentees to receive mentoring [11, 31]. Mentorship programs for women researchers within the SRHR field can help early career researchers to build their skills and promote professional development while increasing research output particularly among women researchers in SRHR.

In 2020, the UNDP/UNFPA/UNICEF/WHO/World Bank Special Programme of Research, Development and Research Training in Human Reproduction (HRP) at the World Health Organization (WHO) developed a mentorship programme for early career women researchers in sexual and reproductive health and rights (SRHR) through its HRP Alliance for research capacity strengthening [32, 33]. Briefly, the aim of the programme was to pair early career women researchers from low- and middle-income countries (LMICs), affiliated with institutions that are part of the broader HRP Alliance network, with mid- to senior-level mentors through an open, competitive call for applications. The programme sought to facilitate professional development by offering career advice based on personal skill building, promoting and sharing networking opportunities and offering support in navigating personal and professional life challenges based on the mentor’s personal experience [32]. See **Box 1** for a brief description of the activities included as part of the pilot mentorship programme.

Box 1. Processes and activities included in the HRP Alliance mentorship programme*

**Table:** 

**Activity/event**	**Description**
Competitive call for applications to be a mentor/mentee	Open, competitive call with specific outreach through the HRP Alliance. Applicants submitted expressions of interest via an online application system.
Selection and matching of mentors-mentees pairs	Selection and matching of mentor/mentee pairs was largely based on experience, career stage and field of work.
Launch event	Facilitated discussion for all prospective applicants as mentors and mentees on what is mentoring and what to expect from the programme.
Training session for mentors	Selected mentors were offered tools and resources to support the initiation of their mentorship journey. During the training session, mentors were also offered opportunities for role-playing and practicing using the tools provided.
Training session for mentees	Selected mentees were presented with suggestions and recommendations on how to get the most out of their mentorship relationship.
Support sessions for mentors	Participating mentors brought together to discuss issues arising from mentorship, as well as share challenges and opportunities.
Support sessions for mentees	Participating mentees brought together to network, discuss issues, and problem solve collectively.
Celebration event	Participating mentors and mentees, programme organizers, and other stakeholders participated in an online celebration event to share experiences and successes of programme.
Mentorship meetings*	*Encounters*, *self-directed by each of the dyads*, *to discuss topics of their preference using the tools provided through the trainings*.

*Adapted from Brizuela et al. [32]

The objective of this mixed-methods evaluation was to assess the implementation of the pilot mentorship programme with the goal of understanding participants’ experience with the programme. Moreover, the evaluation aimed to assess how participation in the programme supported early career women researchers’ professional and personal goals and objectives. Through the evaluation we also looked to provide suggestions for future mentorship programmes aimed at supporting women researchers at the start of their professional careers.

## Methods

The pilot programme under evaluation included 13 mentor-mentee pairs and lasted 12 months from the call for applications in November 2020 to the end-of-programme celebration event in October 2021. Initial training of mentors and mentees was held in January 2021, with matching being finalized in February 2021 and programme activities commencing thereafter. During the yearlong programme, monitoring of the implementation was undertaken through short surveys after the initial training programme and the ongoing support sessions held throughout and after the end of the programme. For this evaluation, we implemented a convergent parallel mixed-methods study design [34] where we used both quantitative and qualitative methods through an online survey and semi-structured interviews. We used the quantitative data to triangulate with the qualitative data at analysis stage, such that the survey responses helped support the themes emerging from the qualitative analysis [35]. Free text responses from the survey were coded and analysed qualitatively. All 26 participants to the pilot mentorship programme were invited to participate in both the anonymous online survey and the in-depth interviews through email. Only those who responded in the affirmative were recruited into the study in March 2022. The online survey was open between April and May 2022 and the in-depth interviews were held throughout May 2022. The study was reviewed and approved by the WHO Ethics Review Committee (18 January 2022, ID: ERC.0003705).

### Qualitative data collection and analysis

Two separate qualitative semi-structured in-depth interview (IDI) guides were developed to conduct the interviews: one for mentors and one for mentees. The guides aimed to explore participants’ experience with the programme, expectations for the programme, perceived successes and achievements, and recommendations for improvement of future iterations of similar mentorship programmes. These interview guides were pilot tested during the training of interviewers and revised and finalized accordingly. Signed informed consent forms were obtained before each IDI and all participants were provided with an information sheet that included details about the study. Interviews were conducted online in May 2022 by three members of the research team (MA; PhD and PM; PhD scholar led, JJC; DrPH observed) using Zoom (Zoom Video Communications Inc., v5.10). Zoom, and other Voice over Internet Protocol (VoIP)-platforms have been proven effective methods for collecting qualitative data, especially during periods of restricted mobility linked to the COVID-19 pandemic [36].

Qualitative interviews were held in English and recorded through Zoom. A note taker also collected the notes during the interview. The average time duration of the interviews was 45 minutes. Audio files were transcribed verbatim by external professionals after all direct identifiers had been removed. The transcripts were not shared with participants for their comment, feedback, and correction. Only the research team had access to participant identifiers and these were kept separately from audio files. Select socio-demographic data were collected at the start of the interviews but were saved in a separate file from the transcripts. All eleven transcripts were checked for quality by two researchers (MA and PM) through listening to the audio files while checking the transcripts. Both quantitative and qualitative results have been reported using the STROBE checklist and the COREQ checklist for observational and qualitative studies respectively.

A preliminary analytical framework was devised encompassing eleven distinct themes to guide the evaluation interviews. These themes covered: the definition of mentorship; motivations for applying to the programme; anticipated programme outcomes; prior experiences with mentorship; interactions within the HRP mentor/mentee framework; the nature of post-programme relationships with mentors/mentees; standout features of the programme; challenges encountered during the programme; perspectives on early-career female researchers; the overall impact of the programme on participants; and recommendations for its further refinement and enhancement. Deductive coding was applied iteratively with each interview to then map these codes to the pre-existing framework by using Microsoft Excel (©2012 Microsoft Corporation). We used inductive methods to further refine the codes and develop themes to best reflect the data obtained from the interviews with the new insights gained by three co-authors (MA, PM and VB). Codes emerging from the data were collaboratively developed and categorized into themes according to their similarities or closeness to similar topics. Developed codes were independently applied to the interviews and researcher notes. Coding was agreed upon by all researchers until all codes and themes had been identified. This process was iterated until we were able to condense the eleven themes into the six final themes.

### Quantitative data collection and statistical analysis

We developed a self-administered survey that was implemented online using Lime Survey (LimeSurvey Community Edition Version 3.28.52). The survey questionnaire contained a combination of multiple choice, ranking, Likert scale, dropdown, and open-ended questions. The questionnaire was expected to be completed in about 25 minutes, depending on skip patterns. All survey responses were anonymized, participation was voluntary, and no questions were mandated, meaning participants could skip questions or abandon the survey at any time. All participants were required to consent to participate before they started completing the survey.

The surveys were distributed via email to all the 26 mentorship program participants using the online platform functionalities; reminders were sent on a fortnightly basis. Questions sought to gather information on participants’ impressions of the programme, suggestions for improvement, as well as whether the mentor-mentee dyads had continued to meet and how these meetings had impacted their professional careers. Some of the questions were specific to mentors or mentees, while the majority were adapted to be relevant to both (e.g., Question: How has the continued mentorship impacted you? Answer options: My mentor has broadened my network/by broadening my mentee’s network). The survey remained open between March and May 2022, approximately six months after formal programme ending.

We used descriptive statistics to calculate frequencies, percentages and means, using Microsoft Excel 365. At data analysis stage, we coded free-text answers from the survey together with the qualitative data from the IDIs, but otherwise analysed qualitative and quantitative data separately and converged our findings during interpretation of results. Open-ended questions were re-coded as applicable and free-text responses were analysed and coded using the framework developed for the qualitative component of this evaluation. We only included in the analysis responses of participants who completed the entire survey, regardless of whether all questions were answered.

### Reflexive statement

The research team was comprised of three WHO/HRP professionals (JC and VB, both female with senior support from AT, female) and two research volunteers affiliated to HRP Alliance network institutions (MA and PM, both male) based in two lower middle-income countries in Africa and Eastern Mediterranean regions; PM was completing his PhD with support from the HRP Alliance. The research team had mixed-methods research experience in sexual and reproductive health in high-, middle-, and low-income countries. JC, AT and VB had been involved in the development and implementation of the mentorship programme and came into the project with *a priori* positive experiences with mentorship provided by women. While JJC observed most of the interviews, in support of MA and PM, neither VB nor AT led in the qualitative data collection. The involvement of two independent researchers (MA, PM) significantly reduced any influence in data collection, analysis and interpretation of results. Furthermore, the interview guides were also independently reviewed by MA and PM who were not involved in the design and implementation of the mentorship program. The evaluation of this programme aimed to offer recommendations for subsequent mentorship initiatives supporting the professional growth of early career women researchers. Several of the team members, having either served as mentors or benefitted from mentorship in their own careers contributed to the development of this programme, drawing from insights gained from their past mentor-mentee interactions.

## Results

From a total of 26 programme participants, this analysis includes responses from 19 (73%) individuals who responded to the end line survey (12 mentees and 7 mentors) and 11 (42%) qualitative interviews (7 mentees and 4 mentors). Completion of the survey took an average of 26 minutes (range 13–53 minutes) while the interviews lasted on average of 45 minutes. Since the surveys were anonymous, it was not possible to tell whether qualitative respondents were the same people surveyed.

**Table 1** summarizes sociodemographic characteristics of study participants. Most participants to this evaluation were mentees (12/19 survey respondents and 7/11 interviewees) and had doctoral degrees (13/19 survey respondents and 7/11 interviewees). Most of the survey respondents were academics (10/19) whereas most of the interviewees were researchers (9/11).

**Table 1:** Participants’ sociodemographic characteristics (N = 19).

Characteristic	Survey participants (N = 19)	Interviewees (N = 11)
Participated as…		
Mentor	7	4
Mentee	12	7
Age		
25–34	3	2
35–44	7	5
45–54	8	4*
55+	1	N/A
Region^α^ of residence		
Americas	5	6
Africa	5	2
Eastern Mediterranean	5	2
South-East Asia	1	-
Western Pacific	3	1
Highest academic degree		
Bachelors	-	1
Master’s	5	3
Doctoral	13	7
Other	1	-
Job title/occupation		
Researcher^§^	6	9
Academic^¶^	10	-
Healthcare provider	2	2
Other	1	-

*Additional categorization of age above 45 years was not provided to interviewees

^α^Regions determined as per WHO categorization: Americas (AMRO), Africa (AFRO), Eastern Mediterranean (EMRO), Europe (EURO), South-East Asia (SEARO), and Western Pacific (WPRO). Countries included in each region can be found at: https://www.who.int/about/who-we-are/regional-offices

^¶^Includes lecturers, professors (assistant, associate, full), and course instructors

^§^Includes researchers and post-doctoral fellows

### Quantitative data

Most survey respondents, 14/19 (74%) stated they ‘*liked a lot’* the participation in the programme and found the training *useful* (3/19, 16%) or *very useful* (16/19, 84%). Most survey respondents had plans to continue meeting with their mentor/mentee after programme end (14/19, 74%), yet few had (8/19, 42%) at the six-month post-programme evaluation mark. Scientific expertise of mentors was ranked as the most important quality in an ideal mentor. **Table 2** summarizes some of the findings from the survey.

**Table 2:** Participants’ reported experiences with the mentorship programme (N = 19).

Responses	n/N	%
Participated in the initial training programme	19/19	100
Training was sufficient to understand the basic concepts behind mentorship	18/18	100
Topics learned during the training		
*What mentorship is*	16/19	84
*How to be an effective mentor/mentee*	14/19	74
*Use of tools for mentorship*	18/19	95
*How to structure mentor/mentee meetings*	14/19	74
*Difference between mentorship and supervision*	18/19	95
*Benefits of a solution-focused approach*	11/19	58
Content of the training was useful during the mentorship you received/provided		
*Very useful*	16/19	84
*Useful*	3/19	16
Prior experience with mentorship programmes	6/19	32
Participation in support sessions (circles)	3.5/4	88
Importance of having a female mentor		
*Very important*	6/19	32
*Somewhat important*	1/19	5
*It depends* ^*^	5/19	26
*Not at all important*	7/19	37
Preferred to have selected mentor/mentee	2/18	11
Topics discussed during mentor/mentee meetings		
*Research technical skills*	7/19	37
*Professional development*	13/19	68
*Research production*	10/19	53
*Networking*	6/19	32
*Personal life*	14/19	74
*Work-life prioritization*	14/19	74
*Power dynamics at workplace*	6/19	32
Frequency of mentor-mentee meetings		
*Twice a month (max*. *16 meetings)*	7/17	41
*Monthly (max*. *8 meetings)*	5/17	29
*Ad-hoc*	5/17	29
Frequency of mentor-mentee meetings considered sufficient	14/19	74
Plan to continue to meet with mentor/mentee	14/19	74
Continued to meet with mentor/mentee	8/19	42
Professional progress since participating in the programme		
*Published peer-reviewed articles*	9/12	75
*Applied for grants*	4/12	33
*Received a promotion or special recognition at work*	5/12	42
*Graduated or special recognition during studies*	4/12	33

*depending on: culture, possibility of opening up and feeling comfortable, on individual characteristics of the mentor

### Qualitative data

From the original eleven themes in our framework, a refined list emerged, consolidating the topics into six distinct themes. These final themes are: views on mentorship; motivations and expectations for participating in the programme; most valued aspects of programme’s implementation; challenges faced within the programme; perceived long-term benefits of the programme; and suggestions for its enhancement. **Table 3** offers a concise summary of these main study themes and their associated findings.

**Table 3:** Study themes and brief findings.

Themes	Findings
Views on mentorship	• Unclear distinction between supervision and mentorship amongst mentees prior to the programme. Participation in the HRP Alliance mentorship programme allowed for clear differentiation between supervision and mentorship. • As a result of participating in the programme, participants started viewing mentorship as a supportive, evolving, and inspirational two-way relationship where benefits were seen by both mentors and mentees
Motivations and expectations for participating in the programme	• Programme offered by a renowned institution (WHO) • Opportunity to network and enhance mentoring skills • Opportunity to become better mentors
Most valued aspects of the programme’s implementation	• Positive engagement between mentors and mentees • New skills gained • Mentorship tools provided • Support sessions (mentor-mentee circles) highlighted as opportunities for sharing and networking by mentors and mentee alike • Support sessions were attended by almost all participants with the majority rating them as very useful • Support sessions indicated as a ‘‘must-keep feature in future programme iterations • Female-centeredness aspect influenced most participants to apply for the programme • Female mentors were keep to support fellow female researchers by offering mentorship • Female mentees felt having a programme for women was beneficial
Challenges faced within the programme	• Time/scheduling conflicts and issues with internet connectivity • Overburdened with work • Language challenges between mentor and mentee pairs • Participants mentioned needing more guidance on programme features, including the objectives and recommended meeting frequency • Having a specific deliverable to work towards was seen as a positive goal that could also benefit participation and engagement between mentors and mentees
Perceived long-term benefits of the programme	• Intangible benefits such as being encouraged to achieve career goals, receiving appropriate guidance on career, motivation for success, and skills on leadership • Tangible benefits such as mentees improving their skills to become mentors, their communication skills, as well as supporting their career development (scientific production, career progression) • Mentors felt that their ability to communicate with student mentees was enhanced through participating in the programme
Suggestions for its enhancement	• Provide seed funding to support joint research work between mentors and mentees • Provide additional technical support to mentees for improved research skills • Possibility for in-person meetings/encounters to support networking

### Theme 1: Views on mentorship

The qualitative analysis of the IDIs and survey free text reveals that before joining the programme, some mentees equated mentorship with supervision. Yet, after participating, they began to discern the nuanced differences between the two. Nonetheless, many emphasized the value of having a mentor with strong technical expertise.

*Mentorship for me in the beginning was more like a supervision, and I wasn’t very aware of the differences between them. After attending this programme, mentorship for me is not more than supervision but is a different kind of supervision like you are providing supervision of other things. (Mentee–IDI)*

Several mentees highlighted that the HRP Alliance mentorship programme stood out distinctly from other programmes they had previously encountered. Overall, both mentors and mentees expressed that they came to perceive the mentor-mentee relationship as a mutually beneficial partnership, fostering professional growth for both parties through both formal and informal means.

*Mentorship is like having guidance and advice from somebody who you can trust*, *who you can connect with and it’s not every time you are looking for a professional person*. *I think a mentor is a person who keeps you motivated and not judge rather inspires (you) to be a better version of yourself*. *(Mentee–IDI*)

*Being able to guide and support a mentee to navigate their professional and academic life guided by your knowledge and experience. (Mentor–survey)*

Particularly, mentors felt that mentoring other professionals also strengthened their own skills and professional networks.

*When I think of mentorship, I’m thinking of growth of both mentors and mentees. I think mentorship is more than a relationship that focuses on skills development and career growth. (Mentor–IDI).*

### Theme 2: Motivations and expectations for participating in the programme

Participants cited various reasons for applying to the mentorship programme. These included the programme’s affiliation with a credible and reputable organization, the World Health Organization (WHO), and its focus on career progression and skills development for mentees. Additionally, the opportunity for networking was underscored as a significant incentive for application. Some mentors were drawn to the programme with the aim of refining their own mentoring skills, while others were motivated by the programme’s emphasis on supporting women.

*To improve my capacity in mentoring and participate in an international network. (Mentor–survey)*

*We have a mentoring programme at my own university, and I felt that [participating in the HRP Alliance programme] will strengthen my mentoring experience, to see what it was like outside of my institution. Maybe I could learn something better from the resources that were out there (…). So, you know the first thing I thought was I just wanted to be part of work being done by a credible organization and secondarily it would benefit me. (Mentor–IDI)*

*The opportunity to have a mentor to help me out on this research and also on my career choices. I was looking also for increase my network not only for mentees but also for the mentors, see other people, see other opportunities, maybe find a new way to collaborate… (Mentee–IDI)*

### Theme 3: Most valued aspects of the programme’s implementation

Overall, participants conveyed favourable feedback regarding the programme, stating that it largely met their expectations, as reflected in both the surveys and interviews. Mentees interviewed expressed that the programme facilitated a constructive learning environment encompassing both research and life experiences. Additionally, some highlighted the effective pairing between mentors and mentees. The support sessions, referred to as “mentor-mentee circles” were particularly praised as the programme’s standout feature. Moreover, the programme’s emphasis on female-centeredness was cited as a significant factor influencing participants’ decision to apply.

*We have a good relationship. I think we discussed a lot of things, not always related to the research, usually it was more about confidence, how to put my research out there, how I could adjust my moment in life with the goals I have further. It was really nice! I really enjoyed it. (Mentee–IDI)*

*…the experience was extremely positive (…) it seemed like that matching process had been done so rigorously that from the onset it was like ‘match made in heaven’. So we kind of got on and we were able to immediately identify the similarities that both mentor and mentee had in common. (Mentee–IDI)*

On the other hand, mentors highlighted the benefit and use of the tools provided during the training session, as well as throughout the programme implementation. Some mentors even mentioned using these tools for the development of mentorship programmes at their institutions. A majority of mentors also indicated that they gained new skills in mentorship while mentees noted that they were able to refine other key skills such as writing and time management.

*So the opportunity to think and to learn tools that maybe I had already used or not was interesting… I think it changed me a lot because I think it mostly enhanced my ability of communicating. So I started using the tools with my students in my meetings and I am currently, at the university, the coordinator of the post graduate course. (Mentor–IDI)*

*So because (my mentor) taught me really good skills and time management skills and writing skills so I used to hear and work on her suggestions. So I think this was the impact of the mentorship program. (Mentee–IDI)*

The support sessions, referred to as “circles” during the programme, were singled out by many as its most outstanding feature. Survey data showed that a majority of the participants attended at least three of the four sessions. Notably, almost all survey respondents (17/19) deemed these support sessions beneficial.

Furthermore, participants in the qualitative interviews indicated that the support sessions helped them to keep track of the progress of the mentoring relationship as they would use these sessions to share common problems in their mentoring relationship and find solutions to these challenges as a group. The support sessions also brought together mentees on a common platform where they could share challenges they were experiencing.

*Best thing is the peer circle that we used to have. (…) because in that we used to have discussion groups and we used to discuss our problems, share experiences with the mentors and there was one-to-one communication with other mentees, some of them have same common problems, some of them were doing really well so we used to take advices from them as well and at the end of the group activity we used to connect through WhatsApp or through chat for informal discussion and chit chat (laughing). (Mentee–IDI)*

*I think the meetings where we had the first contact with the OSKAR [Outcome; Scaling; Know-how; Affirmation/Action; Review] tools, the group exercises. I liked the group exercises, going back with small groups and then talking to everyone together. Yeah I miss the mentor group meetings. (Mentor–IDI)*

Participation in the support sessions were also credited with building relationships amongst mentees.

*What I thought now is that I could have more relationship with other mentees because I know with the mentee circles they were really nice, we were always talking to each other, we were splitting classrooms and I know many other people from the other countries and other mentees. (Mentee–IDI)*

It emerged from the qualitative interviews and the open-ended survey that the aspect of female-centeredness influenced participants to apply for the programme. Reasons ranged from female mentors being keen on supporting fellow female researchers to grow in their professions and paying forward mentorship they themselves had received from fellow female mentors.

*I myself benefited from good mentorship actually from female mentors and in general have been lucky to have the education and opportunities that I’ve had and thought this could be a good opportunity to share some of those skills and experience with someone else (Mentor–IDI)*

Some mentors mentioned that because of participating in a female-centred mentorship programme, their understanding of the complexities that female researchers face improved and intimated that due to the unique and specific challenges faced by female researchers in their careers and life, it was helpful to have a programme specific for women. In fact, most participants noted on the positive aspect of having a programme specifically designed for women; having women mentors was seen as something positive and important in understanding the specific challenges faced by women.

*I don’t think it’s important to be the same sex, but I think it’s helpful to have a programme specifically for females because of the specific challenges women face in research and academics (Mentor–survey)*

*I think that definitely there is the continued need for female supported development. I think that in the academic arena we cannot ignore the fact that as females we are quite not disadvantaged, but we…. the equity still is not there in relation to male early researchers and that is historical so it is not ever going to be remedied overnight. So for those reasons I think that having particular spaces for females to develop in this way, definitely is still needed and I think it is still as important for the transformative processes that we wish to see in institutions (Mentee–IDI)*

Mentees particularly also mentioned that having women-only mentors enabled them to connect emotionally to their mentors and just be ‘women.’

*And then I think you know, that just the opportunity to feel and be as emotional as we are as women you know, excitement, fear, anger and frustration around how we grapple with the other challenges was just phenomenal, we could just be women, we could be excited and cry in our smaller groups you know, be expressive in a way that I think still it did not take away from our professionalism. (Mentee–IDI)*

### Theme 4: Challenges faced within the programme

Reported challenges encompassed issues related to scheduling amidst demanding workloads. These workloads often involved travel to various research sites for both mentors and mentees, compounded by the struggle for stable internet connectivity. While the programme’s remote design facilitated connections between mentor and mentees from different global locations, it also posed difficulties in coordinating meeting and attending circles due to time zone differences. Furthermore, the absence of clearly defined tangible outcomes was noted. Participants suggested that specifying concrete deliverables might have further enhanced the experience for both mentors and mentees.

*I travel a lot and sometimes it is quite challenging to attend the (circles) when I do field work. Sometimes, I feel that I missed some important meetings and insights due to my field work. (Mentor–IDI)*

*So initially I really struggled to get time for the first meeting so because we have a lot of scheduling conflicts and time issues because obviously I was the mentee and I was really desperately looking for the mentorship and the meetings but (my mentor), she is the head of a university and she has a lot of other responsibilities so that’s one thing I struggled initially in the first few weeks after the first proper meeting with (my mentor). (Mentee–IDI)*

*I think that network did not allow me to really connect so well, and I felt that maybe I needed the recording of the (support) session to be able to help me catch up with what I could not get. (Mentee–IDI)*

Some mentors and mentees indicated that there were a few communication challenges given the programme was implemented in English but the matching had not taken into consideration the native language of mentors and mentees. However, this did not seem to affect the programme success.

*And also some kind of, I think there was very little bit of language barrier as well so I think that also played a part for not making the relationship last. I am not sure that much but it is just my opinion. (Mentee–IDI)*

*It is possible maybe that someone who either spoke her language or who had done more work in that country may have been a better match but I don’t think it would have been a big difference. (Mentor–IDI)*

The absence of distinct products or outputs from the mentorship programme emerged as a concern. Both mentors and mentees had anticipated working towards a tangible goal by the programme’s conclusion. Additionally, participants highlighted the importance of establishing predefined objectives tailored for each mentor-mentee pairing.

*One thing that I also mentioned earlier is that there was no predefined objectives…it would be beneficial if we had some kind of objective meeting schedule and timings written out by the HRP Alliance because initially we struggled for this to sort out what would be the learning objectives, what would be the outcomes and there was no outcome mentioned so everyone joining this programme had different outcomes and different experiences. (Mentor–IDI)*

*So my experience was after first initial few weeks then I started to gather that okay this is what I am supposed to do, in each meeting we will be discussing this and then after first few weeks I think there was more clarity which was not in the initial few weeks because we didn’t have any objective from the HRP and also so there was a brief guideline but was not a very strict guideline about the frequency of the meetings so both of us were very confused that how frequently we should meet. (Mentee–IDI)*

Some mentors and mentees also expected that they would work with their other half towards pre-defined milestones within a prescribed timeframe. Relatedly, the duration of the programme was also mentioned as insufficient to work on tangible outputs.

*Generally, my expectation was to be sort of paired with a motivated researcher from a lower middle income country female scholar who is interested in advancing their career and that there was sort of, there will be some formal components whether they were meetings or trainings or milestones or something and there will be a timeline associated with it and I think other than that I didn’t know where the details would end up being (Mentor–IDI)*

*I think so if your programme can increase the duration, the duration was limited and they could actually come up with tangible outcomes like you know, publishing paper. (Mentee–IDI)*

### Theme 5: Perceived long-term benefits of the programme

In the interviews, mentees and mentors reported numerous benefits, both tangible and intangible, that they felt were in part a result of participating in the programme. For example, intangible benefits for mentees included being more mindful of their work-life balance and believing, based on encouragement from their mentors, that it is possible for women of any socio-cultural background to focus on their careers and family responsibilities at the same time. Mentees also reported appreciating the guidance and motivation they received from their mentors early on in their careers.

*It is really very important to have I think correct guidance at early stage of the career because right now we are in learning phase. So if we learn correctly, if we have good direction, guidance then we can actually excel in much better way rather than just struggling for correct opportunities, correct technical skills. (Mentee–IDI).*

Mentorship was also perceived to play a role in assisting mentees to manage their own expectations, and deal with feelings of rejection in their professional progression (for instance regarding rejected scientific articles) and of perseverance.

*I think first of all, mentorship will, how do I say this kindly, will help them not to have unrealistic expectations. A lot of people and I heard this from a lot of the conversations we had, it is like they want to jump and go so fast and they don’t realize that it takes time to grow. We are still growing even today. (Mentor–IDI)*

*I got disappointed because I had my papers rejected, my mentor was there to encourage me and told me that, ‘Look, we have all been through it, so brace yourself and pick it up’. So I resubmitted those papers and as we speak one of them, I sent revisions and it is at the stage of acceptance. So I think from the programme I learnt to rise up from my fall and then move ahead. (Mentee–IDI)*

Regarding concrete benefits, both mentors and mentees discussed the influence the programme had on enhancing their mentoring and communication capabilities, as well as their career development–spanning career advancement, research output, and networking. For instance, some mentors remarked that the skills acquired during the programme proved invaluable in their dairy interactions with students.

*I think it changed me a lot because I think it mostly enhanced my ability of communicating. So I started using the tools with my students in my meetings and I am currently, at the university, the coordinator of the post graduate course. So I am always looking for the women that I see as mentors now and then I am calling them and talking to them on how we have to be more than supervisors and it is important to acknowledge that role and how to talk to the students. So I guess it did impact a lot in my practice. (Mentor–IDI)*

*I am about to pursue the promotion, work promotion from lecturer to senior lecturer. So preparing that document and like I said, being able to develop a professional development plan for the next five years. So I had two out researchers’ papers, no I had three. Three during the period of the mentorship programme. I think another output has just been the networking and the links of individuals that we have built relationships with. (Mentee–IDI)*

### Theme 6: Suggestions for its enhancement

Many of the recommendations proposed by participants were directly related to the issues raised regarding challenges with the programme implementation (e.g., increased duration) and the request to having a tangible output (e.g., a publication) or pre-defined outcomes for the mentorship relationship. In addition, participants mentioned the possibility to meet in-person as a recommendation to aid in professional networking. Further, some respondents recommended providing financial support in the form of seed funding to support the mentor-mentee pairs to work on joint technical deliverables such as scientific papers and grants. This was supplemented with a recommendation to provide additional research methods technical support to mentees, as a way to best develop their research careers.

*I think that funding has been a major challenge. If you even want to pursue something and you do not have funding to be able to do it, it limits you and so you are not able to pursue it. So, I think if there are small grants available for early career researchers it would also help. (Mentee–IDI)*

*I would suggest offering more technical support in mentoring and research-building capacity. For example, we could have an online platform with textbooks, reports, manuscripts, or practical exercises, dos and don’ts, etc. (Mentee–IDI)*.

*Let’s consider having the final meeting/graduation ceremony to be an in-person event to strengthen and enhance the networks. Both mentees and mentors should be invited for at least 2 days for a short conference with keynote speakers and increase the chances of possible collaborations. (Mentee–survey)*

## Discussion

This paper presents findings from the evaluation of a global pilot mentorship programme for early career women researchers organized by the HRP Alliance at the World Health Organization. Through qualitative and quantitative data, we assessed participants’ experiences with the programme, and how participants perceived that the programme had supported career progression.

The programme was very positively received by most participants, both from the survey and from the interviews. This is in line with what others have found with mentorship programmes in general including those that report on benefits to mentors, defining mentorship as a two-way relationship [12]. Mentors in this pilot programme were offered specific training in mentorship with the goal of strengthening mentoring skills for a successful mentorship relationship, and most of the participants valued this training highly. The decision to include training was based on existing evidence of its need which complements what we know about its importance [37]. Other studies have reported that frequency in meetings between mentors and mentees are important in solidifying the relationship and while there is no clarity regarding what the ideal frequency should be, participants in this programme found them adequate [38]. In general, and relatedly, respondents thought that opportunities for networking and meeting with other mentors and mentees alike were among the most salient aspects of the programme. Others have also found that programmes that focus on professional networks and possibilities for exchanging ideas are constructive [38]. Similarly, the need for guidance and support at the start of a research career [39].

An important characteristic of this programme was the focus on women. This was perceived as a positive aspect both at the time of applying to the programme as well as throughout execution. Despite participants being open to making the programme include people of all genders, they valued the focus on the specific challenges that women face at the start of their career. Specifically, women mentioned issues around gender bias, power asymmetry, and work-life balance, something others have identified in the pursuit of developing female leaders [40]. While support through overall wellness and work-life balance were also identified by others [39, 41], the specific focus on women is important because of the pervasive gender inequality in science and academia [3, 42–44]. It has also been documented elsewhere that women’s contribution to science is often unacknowledged, undervalued and underappreciated [10].

Participants in the programme saw value in the distinction between mentorship and supervision, which they learned about as part of participating in the programme. Although some participants felt that the HRP Alliance mentorship programme should have included specific outputs, generally this did not seem to affect the general success of the program. There is evidence from another mentorship programme that also focused on mentoring elements that did not include specific research outputs and it was positively viewed by participants [37]. Similarly, others have also shown that mentees value mentors’ support to their work-life balance which came through in this evaluation [39]. Furthermore, much of the literature focuses on the importance of having a caring and supportive relationship where guidance and communication are key, similarly to what we found in this evaluation [39, 45]. However, the HRP Alliance mentorship programme had a very specific aim of providing support and skills development as part of researchers’ professional development, but which did not include a specific output or development of research skills. This expectation was part of other mentorship programmes developed for clinical or research students or professionals [39]. And interestingly, participants to this mentorship programme also valued their mentors’ scientific expertise among the highest-ranking characteristic. The intertwined concepts of academic supervision and mentorship seem to continue to permeate expectations of participating in such programmes; even after stating distinction between these concepts, participants still valued scientific and technical aspects highly while simultaneously highlighting the need for supportive and caring mentors as evidenced from survey and interview responses.

This evaluation has some limitations. First, given the focus on building professional skills–often called soft-skills–we were unable to collect hard data on changes in knowledge, practice or behaviour with regards to their academic performance. Second, there is a risk of social desirability biasing the qualitative data, especially since the evaluation was led by individuals involved in developing and implementing the programme. However, the data obtained through the anonymous surveys confirmed much of what we learned through the interviews, making us more confident in our findings. Relatedly, there is a possibility of selection bias; those responding to the survey were likely those more in favour of the programme and similarly, those agreeing to be interviewed were likely to have been those who viewed the programme positively. In addition, since participation in the quantitative survey was anonymous, we cannot link the responses to those of the qualitative interviews as we did not link both sets of respondents. Third, participants included in this evaluation corresponded with individuals who self-selected to take part in the programme and remained active throughout the entire year of programme implementation, meaning these are probably people who are interested in mentorship and open to learning about it. Fourth, we only collected data at one point in time at the six-month mark post-programme end, meaning we don’t have knowledge on longer term effects of the programme. Relatedly, the scaled mentorship programme that the HRP Alliance has led has also incorporated surveys and other mechanisms to collect data throughout and is intending to conduct further surveys with all cohorts of participants [32]. Lastly, this evaluation includes responses from a limited number of individuals that were part of the HRP Alliance pilot mentorship programme so results cannot be generalized to the broader population. However, our findings provide useful insight for others looking to develop and implement such programmes.

Our findings have implications for academics and others providing mentorship to early career researchers, as well as for institutions and researchers looking to develop global mentorship programmes. There appear to be ample benefits to mentorship, especially when planned and implemented in a structured manner. These can be particularly beneficial when they are conceived as a two-way relationship of mutual learning and support, and especially for women at the start of their research careers as they navigate structural gender inequities. The lasting effects of programmes like the one the HRP Alliance has been conducting since 2020 are uncertain; further follow-up and understanding of longer-term effects would be needed.

## Conclusion

Mentorship programmes aimed at early career women researchers in sexual and reproductive health and rights are positively viewed by participants and are perceived as assets in their career development. Mentorship that focuses on providing support, enhancing networks, and lasting relationships have the potential to have lasting positive effects.

## Supporting information

S1 Data (XLSX)
